# The Topology of Pediatric Structural Asymmetries in Language-Related Cortex

**DOI:** 10.3390/sym12111809

**Published:** 2020-10-31

**Authors:** Mark A. Eckert, Federico Iuricich, Kenneth I. Vaden, Brittany T. Glaze, Dyslexia Data Consortium

**Affiliations:** 1 Hearing Research Program, Department of Otolaryngology-Head and Neck Surgery, Medical University of South Carolina, Charleston, SC 29425, USA;; 2 Visual Computing Division, School of Computing, Clemson University, Clemson, SC 29634, USA;

**Keywords:** structural asymmetry, language laterality, topological data analysis, persistent homology

## Abstract

Structural asymmetries in language-related brain regions have long been hypothesized to underlie hemispheric language laterality and variability in language functions. These structural asymmetries have been examined using voxel-level, gross volumetric, and surface area measures of gray matter and white matter. Here we used deformation-based and persistent homology approaches to characterize the three-dimensional topology of brain structure asymmetries within language-related areas that were defined in functional neuroimaging experiments. Persistence diagrams representing the range of values for each spatially unique structural asymmetry were collected within language-related regions of interest across 212 children (mean age (years) = 10.56, range 6.39–16.92; 39% female). These topological data exhibited both leftward and rightward asymmetries within the same language-related regions. Permutation testing demonstrated that age and sex effects were most consistent and pronounced in the superior temporal sulcus, where older children and males had more rightward asymmetries. While, consistent with previous findings, these associations exhibited small effect sizes that were observable because of the relatively large sample. In addition, the density of rightward asymmetry structures in nearly all language-related regions was consistently higher than the density of leftward asymmetric structures. These findings guide the prediction that the topological pattern of structural asymmetries in language-related regions underlies the organization of language.

## 1. Introduction

Significant advances have been made in our understanding of structural and functional cerebral asymmetries [1,2]. Nonetheless, there remains some mystery about the development of structural asymmetries and their significance for brain organization and individual variation in behavior [3]. Specifically, there is limited understanding about the significance of left cerebral hemisphere dominance for some language functions, whether and how structural asymmetries may underlie this specialization, what functional advantages structural asymmetries may confer, and how intrahemispheric organization influences language functions [4].

A defining feature of the human brain is its asymmetric structure, including in language-related brain regions that exhibit leftward asymmetric activation during language tasks [5–7]. Leftward anterior insula asymmetry and rightward superior temporal sulcus asymmetry, for example, have been consistently observed across surface area and volumetric measurement methods where univariate voxel-level analyses were performed in pediatric [8,9] and adult samples [10–13]. While variation in insular asymmetry has been related to estimates of language dominance and asymmetric activation in at least three studies where volumetric asymmetries were examined [14–16], measures of other language-related regions have been inconsistently associated with language laterality [14,16–20]. One explanation for these inconsistent findings is the influence of demographic and cognitive variables that have been related to structural asymmetries.

Sex has been related to structural asymmetries in multiple voxel-based studies [10,13,21], although non-significant results have been reported [8,11], perhaps because sex differences in brain size may explain locally specific sex effects in some samples [22]. The magnitude of sex differences may also depend on age. Males exhibited more pronounced rightward asymmetry in cortical surface area than females, but this effect was most evident in younger participants [13]. Age has also been related to structural asymmetries, and these effects appear to be dependent on sample size, the range of age, and perhaps the method of analysis. For example, increasing age was modestly associated with more leftward asymmetry in superior temporal gyrus cortical thickness and more rightward asymmetry in superior temporal sulcus surface area [13].

Variation in structural asymmetries has also been related to oral language abilities, although inconsistently. An early imaging study demonstrated higher verbal comprehension (VIQ) when a left parieto-occipital region was larger than the right in a reading disability sample [23]. Leftward posterior superior temporal gyrus asymmetry was also significantly associated with an estimate of VIQ [9]. Better expressive and receptive language has been observed in children with less rightward gray matter volume asymmetry of the pars triangularis (Brodmann area 45), which appeared largely due to the volume of the right pars triangularis [22]. In addition, change towards more rightward pars triangularis cortical thickness, due to reduced left cortical thickness, was associated with larger gains in language skills in children [24]. This smattering of associations with asymmetries across the cortex involved different methods and different sampling approaches. Still, there have been relatively few consistent findings, and some non-significant results have been reported [25].

The structural asymmetry findings described above all involved gross morphometric approaches where asymmetry values were averaged across a relatively large region of the brain, or the analyses involved voxel-level asymmetry comparisons. Structural asymmetries, at least volumetric or surface area asymmetries, have unique spatial distributions and exhibit three-dimensional structure. Topological data analysis is well-suited for characterizing the morphology of brain structure asymmetries. That is, spatially complex structural asymmetries can be measured with topological approaches that distill this complexity, as well as some redundant information at the voxel-level, to a minimal but precise representation of the asymmetry.

Persistent homology, the most widely adopted tool in topological data analysis is a mathematical tool rooted in algebraic topology, which can be used to characterize the persistence of a structure across a given dimension. This includes voxel values in images [26] where a structure has voxel values with a local minima and local maxima. In the case of voxel-based structural asymmetries, a leftward asymmetry in a left hemisphere language-related region would be represented with a range of positive values defining a hyperintense cluster of voxels. Likewise, rightward asymmetries would be represented in the same left hemisphere region with a range of negative values defining a hypointense cluster of voxels or a void. Persistent homology is used to measure the local maxima and minima of the contrast values that define leftward or rightward asymmetries. An example of the persistent homology measure of leftward and rightward asymmetries is presented later in the Methods.

The overarching goal of this descriptive study was to characterize the topology of deformation-based or volumetric asymmetries within language-related areas that exhibit asymmetric patterns of activity. Specifically, analyses were performed to examine the extent to which topological asymmetries were observed in 18 core language-regions, as defined by a functional language laterality atlas [6]. Sex, age, and VIQ associations with the topological asymmetries were examined to determine the extent to which these associations would replicate previous findings [13] and, more generally, to characterize the extent to which these factors explain some asymmetric variation in language-related brain regions.

## 2. Materials and Methods

### Participants.

Retrospective data from 212 children (mean age = 10.56 years, range 6.39–16.92; 39% female) across 10 research sites were included in this study. These data were collected as part of a study to develop methods for multi-site retrospective neuroimaging studies and are part of a Dyslexia Data Consortium database (www.dyslexiadata.org). This normative study focused on children with typically developing language skills. Informed consent and institutional approval for the original research was obtained at each institution. The data were de-identified prior to data sharing, which was approved by the contributing institution and the Medical University of South Carolina Institutional Review Board. Data from a subset of these children has been reported previously [27–30].

### Imaging Data and Image Processing.

The T1-weighted image acquisition parameters for each research site are listed in Table 1. The images were denoised [31], bias field corrected using the Statistical Parametric Mapping (SPM) non-uniformity (bias/apply) functions, and then rigidly aligned to the MNI 152 T1 1mm template using the SPM coregistration function. These images, and their left-right flipped copies, were used to create a study-specific symmetric template using Advanced Normalization Tools (ANTs v 2.0) [32]. The native space images were also segmented using the SPM new segment function so that a total brain volume estimate could be obtained from the sum of the gray and white matter images.

A two-step ANTs normalization procedure was performed where an optimal symmetrical template was created without regard for how much each image would be warped or distorted. This template was used in step 2 to more conservatively align the individual images to the optimal template to prevent over-regularization or grossly distorted anatomical features within each participant’s image. Specifically, 1) ten normalization iterations were initially performed to create the optimal template where the mean of the images from the preceding step served as the normalization target for the next iteration [normalization parameters—iterations 1–9 (30 × 90 × 20 × 12); iteration 10 (30 × 90 × 30 × 20); across iterations: cross-correlation metric (mm radius = 4.00); SyN (2, 1, 1); Gaussian regularization (4.00)]. The optimal symmetrical template was then 2) used as a target for a series of three normalization iterations that varied in the magnitude of volumetric displacement, as described in [8] [normalization parameters—iteration 1 (50 × 1 × 1): cross-correlation metric (mm radius = 4.00), SyN (1.00), Gaussian regularization (3.00); iteration 2 (1 × 50 × 1): cross-correlation metric (mm radius = 2.00), SyN (0.75), Gaussian regularization (2.00); iteration 3 (1 × 1 × 40): cross-correlation metric (mm radius = 1.00), SyN (0.50), Gaussian regularization (1.00)].

The warping parameters from each of the three normalization iterations were combined to generate warps that align each native space image to the symmetrical template. These warps also characterized how much volumetric displacement was necessary to move a voxel into the template space. This volumetric displacement was represented by the Jacobian determinant, which was obtained using the ANTSJacobian function that included the linear and nonlinear displacements and was log-scaled. Jacobian values representing the warping of relatively large structures had larger values compared to relatively smaller structures. The Jacobian images were then smoothed with an 8 mm full width at half maximum Gaussian kernel for consistency with previous voxel-based studies [21,33]. Because the images were log-scaled, each Jacobian image could be subtracted by its left-right flipped copy to create a standard asymmetry image where hemispheric differences are scaled by the mean volumetric displacement of the left and right hemispheres. An overview of these image processing procedures is presented in Supplementary Figure S1.

The Jacobian asymmetry results presented later can be interpreted in the context of other voxel-based studies of gray and white matter asymmetries where Jacobian modulation of the gray or white matter voxel values was performed. That is, the voxel-level asymmetries with this Jacobian or deformation-based approach are very similar to the voxel-level asymmetries involving specific tissue types [8]. Given the similar pattern of results between voxel-based gray matter volume asymmetries and surface area asymmetries [9,12], the current volumetric deformation approach was also expected to yield results that are similar to surface area asymmetry results.

The imaging parameters in Table 1 were obtained from de-identified DICOM information when available, otherwise from related manuscripts or image header information, which is why flip angle information is unknown for one site. The relatively long repetition times (TRs) above were used for inversion recovery acquisitions (inversion times: site 2 = 900 msec; site 5 = 640 msec). Site 4a,b data were obtained for 2 different studies from 1 research site. GE—general electric; TR—repetition time; TE—echo time.

### Regions of Interest.

The [SENSAAS; [6]; https://www.gin.cnrs.fr/en/tools/sensaas/] was used to define language-related ROI. The SENSAAS is based on an atlas of homologous interhemispheric regions that exhibited common patterns of resting state activity [34]. This includes 18 core regions that exhibited leftward activation asymmetries, which appeared to be essential for sentence processing and where lesions can produce language deficits [6]. The SENSAAS ROI are in MNI coordinate space, so the 1 mm T1 MNI template was ANTs normalized into the space of the optimal symmetrical template. The warping parameters were applied to each of the SENSAAS ROI to put them into the same coordinate space as the asymmetry images [Normalization parameters- 1 iteration (30 × 90 × 30 × 20); Gaussian regularization (4.00); SyN (2,1,1)]. To determine the influence of ROI size on the results from the persistent homology measures, the volume of each ROI was determined by summing the voxels in each binary ROI. In addition, the mean asymmetry within each ROI was obtained using MarsBar [35] for comparison to the topological asymmetry data.

### Persistent Homology.

**Figure 1.**
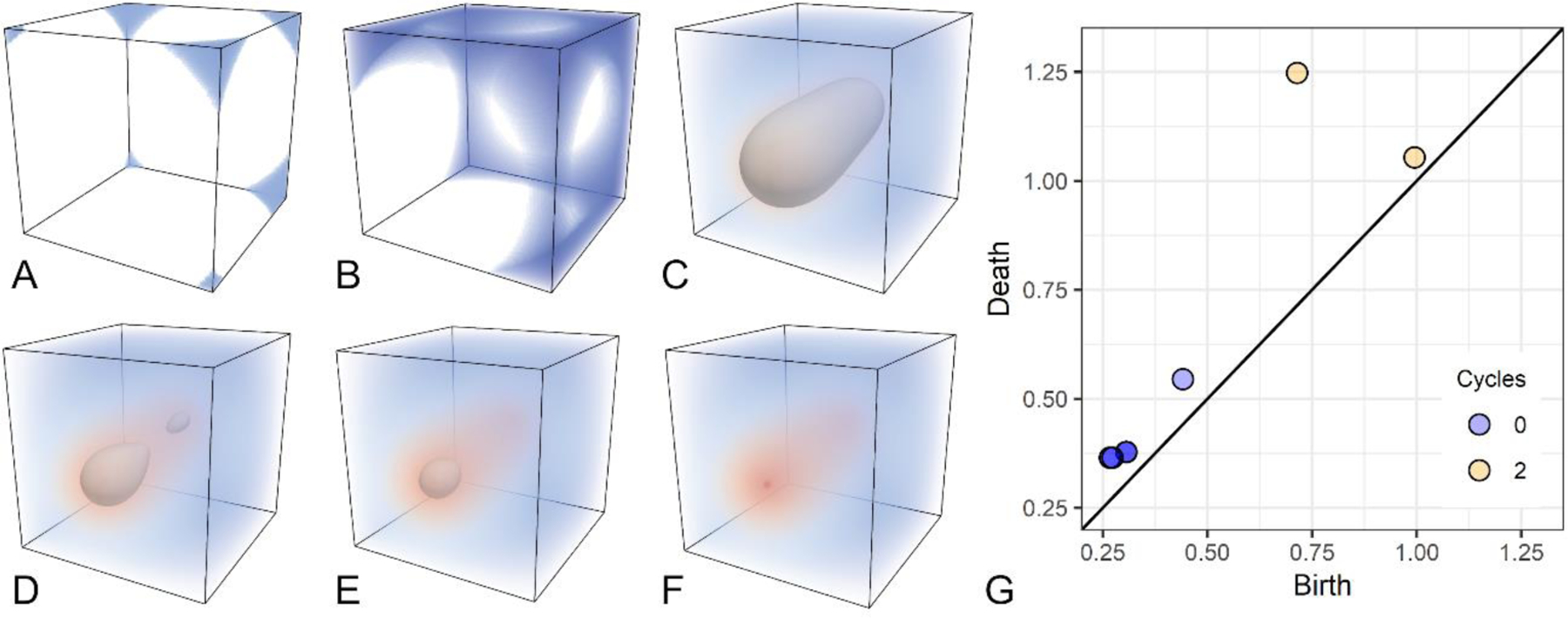
Example of the sublevel set filtration defined on a 3D image and persistence diagram. The intensity of voxels is color-coded according to a diverging (blue-red) color map. Gray surfaces indicate cavities appearing and disappearing in the filtration from (**A**–**F**). (**G**) The 0–cycle and 2–cycle structures identified in the filtration are presented in the persistence diagram where the x and y axes indicate the contrast values corresponding to the birth and death of each structure. Note the short lifespan of the seven 0–cycle structures with low contrast values in A are clustered at the bottom of the identity line in (**G**). Also note the two 2–cycle structures with higher contrast values are shown in (**G**) with one 2–cycle structure having a longer lifespan (range of contrast values) based on its distance from the identity line.

Persistent homology is a widely used tool, grounded in algebraic topology, to analyze shape features by means of a filtration. Please see [36] for the original description of this approach, which we formalize as follows. Let Γ represent a cubical complex (i.e., the image grid), the filtration is defined as a sequence Γˆf = {Γˆi |0 ≤ I ≤ r}, such that Ø = Γˆ0⊆Γˆ1⊆···⊆Γˆr = Γ. When working with images, a filtration is naturally defined as the sequence of sublevel sets of Γ. Intuitively, a filtration corresponds to a growing object obtained by increasing an intensity threshold to points in a 3D image (i.e., voxels), as shown in Figure 1. Voxel intensity is color-coded according to a diverging (blue-red) color map. Low-intensity voxels are introduced first in the filtration, generating seven components (blue regions in Figure 1A). As voxels with higher intensities are added, the seven components merge into one (Figure 1B). Eventually, all voxels on the boundary of the image are introduced, thus creating a cavity (Figure 1C). The border of this cavity is depicted in gray. While shrinking, the cavity splits into two in Figure 1D. The newly born cavity is filled up in Figure 1E. Finally, the last cavity disappears when voxels across intensity values are introduced by the filtration (Figure 1F).

Persistent homology keeps track of all components, or 0–cycles, and hyper-intense objects, also called 2–cycles, which appear and disappear through filtration. Each cycle is characterized by a pair of indices (i,j), or a persistence pair, that describes when the cycle was generated (birth) and destroyed (death), during filtration. It can be shown that the birth of a 0–cycle corresponds to a local minimum in the image [37]. In contrast, the death of a 2–cycle corresponds to a local maximum. In this asymmetry study where ROI were placed in the left hemisphere, the continuum of asymmetries reflected contralateral to ipsilateral asymmetries. 0–cycles corresponded to contralateral asymmetries and 2–cycles correspond to ipsilateral asymmetries. The lifespan of each cycle defines the range of asymmetry values that represents the asymmetric structure.

The Topology Toolkit [v.0.9.8; [38]] was used to compute persistence pairs from each participant’s asymmetry images. Again, 0–cycles characterized voids or contralateral asymmetries, and 2–cycles characterized hyperintense objects or ipsilateral asymmetries in the left hemisphere. For each ROI, we collected all contained persistence pairs. Structures defined by persistence pairs can extend beyond the space of an ROI. For this reason, persistence pairs corresponding to rightward asymmetry (0–cycle) were included in a ROI only if the birth of the structure occurred within the ROI and had a negative birth value to ensure that the structure was rightward asymmetric. Similarly, persistence pairs corresponding to leftward asymmetry (2–cycle) were included only if the peak voxel value or death of the structure occurred within the ROI.

**Figure 2.**
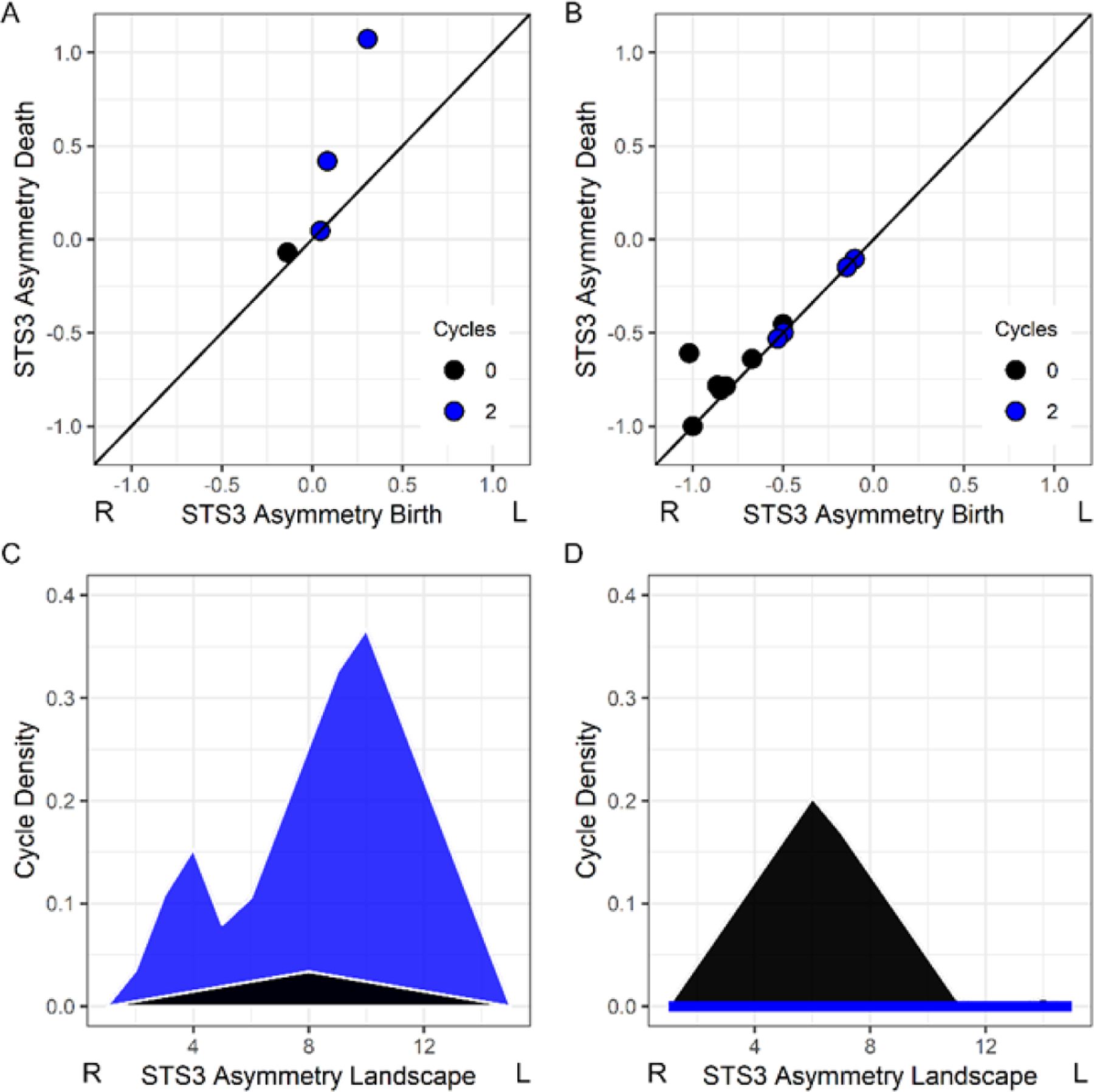
Examples of the persistent homology measures and participant variation for a superior temporal sulcus ROI (STS3). (**A**,**B**) Persistence diagrams of the 0–cycle and 2–cycle persistence pairs, or rightward and leftward asymmetries, for 13.5 year-old male and 11.6 year-old female, respectively. Each circle on the diagonal is typically a very small structure or noise. (**C**,**D**) The persistence pairs from each participant are represented by persistence landscapes (black: 0–cycle; blue: 2–cycle). These landscape data were used to examine varied asymmetries in the ROIs across participants. The R and L indicate rightward and leftward asymmetry, respectively.

Persistence pairs are commonly visualized using a persistence diagram or scatterplot graph representing each pair as a dot with coordinates (i,j), as shown in Figure 1G. The longer the lifespan of a cycle, the more distant the corresponding point will be from the diagonal in the persistence diagram. Figure 2A shows a child’s asymmetry image where there were two leftward asymmetric structures (blue circles) within a superior temporal sulcus ROI. Figure 2B shows another child’s asymmetry image where there was a single rightward asymmetric structure in the superior temporal sulcus and no evidence of a leftward asymmetry.

Persistence landscapes were used to transform the persistence diagrams into a normalized vector space that represents the lifespan (i.e., magnitude of each asymmetry) and density of asymmetries or cycles across intensity values [39]. The transformation from persistence diagram to landscape allows for statistical comparisons across participants who can have a different number of persistence pairs. The R TDA library (v 1.6.5) [40] was used to create persistence landscapes that represented persistence pairs across 15 positions representing the range of asymmetry values in the images. We later refer to statistical effects at a landscape position because the landscape range represents rightward to leftward asymmetries. Figure 2C,D shows the landscapes for the persistence pairs in Figure 2A,B.

The persistence landscape data was not redundant with the mean asymmetry of all voxels within an ROI, because the persistence homology approach distinguishes leftward and rightward asymmetries that would otherwise be averaged across an ROI. In addition, differences could occur because the approach used here excluded asymmetric structures that were not born in an ROI (rightward asymmetries) or that did not die within an ROI (leftward asymmetries). Thus, the mean asymmetry data exhibited varying degrees of association with the landscape data depending on how many rightward and leftward asymmetric structures were within an ROI. For that reason, we examined the relative association strengths between the persistence landscape and mean asymmetries with age, sex, and VIQ, as described next in the Statistics section.

### Statistics.

R (v 3.6.0) was used for all statistical analyses. This included the use of the mice library (v 3.6.0) to deal with the 18% multi-site missingness for the VIQ measure [28,41]. Predictor variables in the imputation model were used to create 10 imputed datasets and included age, sex, research site, and total gray matter and white matter volume. The following behavioral variables were not a focus of this asymmetry study but were also included in the multiple imputation model: (1) real word identification (Letter–Word Identification; mean = 109.13, sd = 12.21); (2) pseudoword decoding (Word Attack; mean = 107.99, sd = 11.00), and (3) reading comprehension (Passage Comprehension cloze task; mean = 105.92, sd = 9.40) subtests from the Woodcock–Johnson IIIR [42] and Woodcock Reading Mastery Tests [43]; and (4) rapid naming (mean = 99.67, sd = 11.57) from the Comprehensive Test of Phonological Processing Rapid Automatized Naming or the Rapid Alternating Stimulus Tests [43–45]. The VIQ measures from each of the 10 imputed datasets were highly correlated. For example, VIQ from imputed dataset 1 (mean = 113.00, sd = 14.09) explained 99% of the variance in the VIQ from imputed dataset 10 (mean = 113.06, sd = 14.11). Thus, a single imputation was sufficient and for this reason, VIQ results described later are reported for only the first imputation.

Nonparametric linear regressions were performed to examine age, sex, and VIQ associations with the persistence landscape data. These analyses included the research site as a control variable for potential differences in image acquisition across sites. Follow-up analyses were performed to determine the extent to which total brain volume could explain sex differences. To limit false positive findings and analyze non-normally distributed landscape data, the lmPerm library (v 2.1.0) [46] was used to evaluate the significance of each predictor variable using the lmp function and the permutation F-test probability method. Significant associations were also examined to determine the extent to which the mean asymmetry within each ROI exhibited the same relationship with age, sex, and VIQ as the persistence data. Here we adapted the test of two independent correlation coefficients [47]. The t-score results from each lmp regression result were converted to r values and we used the degrees of freedom from the regression in calculating the significance of differences between landscape asymmetry and mean ROI asymmetry effects.

We also determined the extent to which there was a difference in the density of 0–cycle and 2–cycle asymmetries within each of the 18 language-related ROI. Each of the 0–cycle and 2–cycle landscapes for an ROI were subtracted, and then the mean of this landscape difference was obtained to produce a metric of the relative prevalence of leftward versus rightward asymmetries in each ROI. A one-sample t-test was then performed to examine the extent to which the differences were significant using Wilcoxon Rank non-parametric permutation testing tests with the exactRankTests library (v 0.8–31) [48]. Finally, standard voxel-based morphometry was performed using the CAT12 Toolbox implementation of threshold-free cluster enhancement (TFCE) non-parametric testing [49,50] with a corrected probability threshold of *p* < 0.05 to demonstrate where there were significant leftward and rightward asymmetries at the voxel-level for comparison to the topological results.

### Data Availability.

Data can be made available with institutional data sharing approvals. Please contact the corresponding author for details, as well as for access to the topological data analysis code used in this study.

## 3. Results

**Figure 3.**
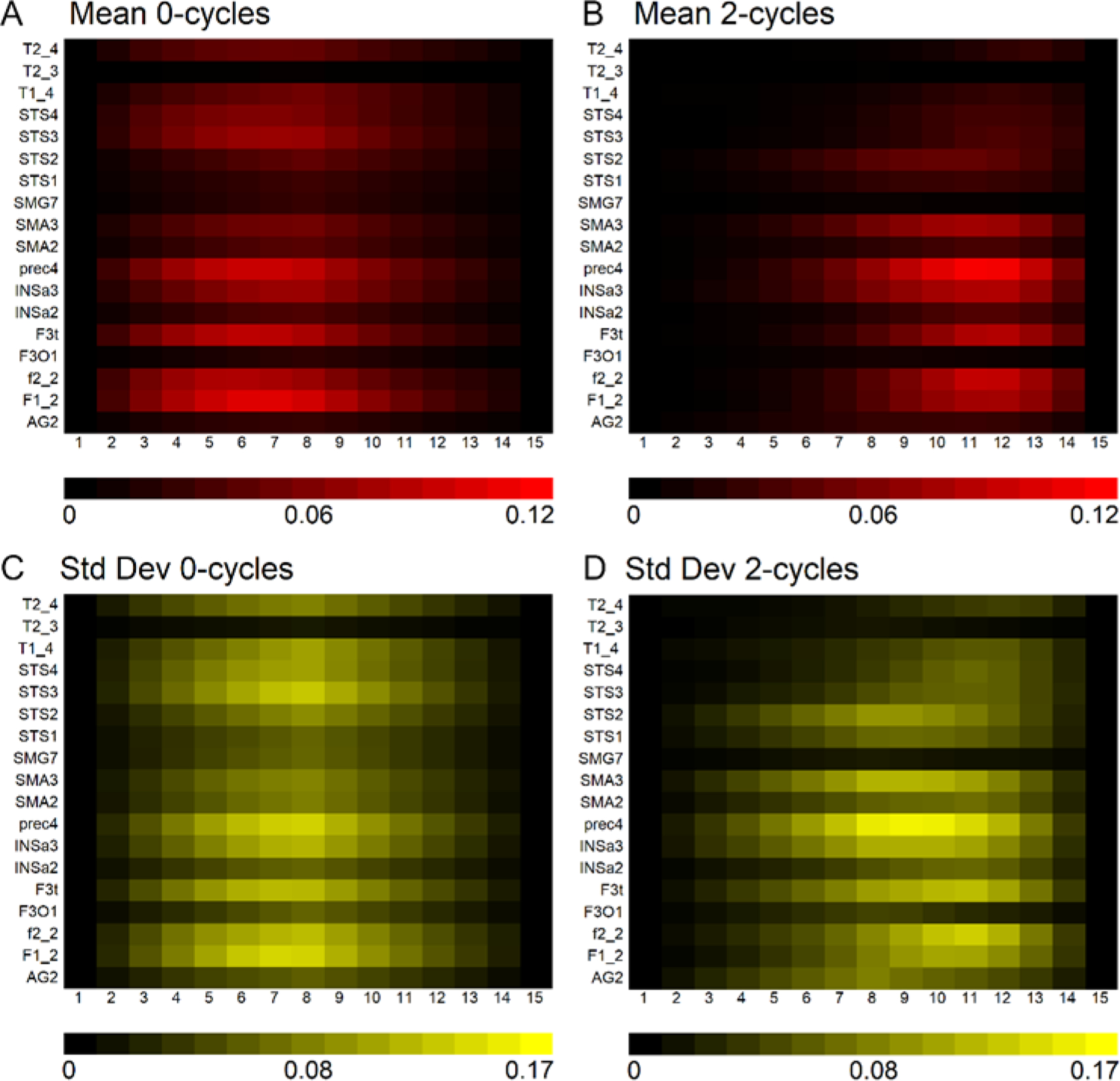
Rightward and leftward asymmetries in the same language-related brain region. (**A**,**B**) Mean 0–cycle or rightward asymmetries and mean 2–cycle or leftward asymmetries. The brighter red regions show where asymmetries were most pronounced or dense across the landscape (x-axis). (**C**,**D**) Std Dev are shown for each of the ROI across the landscape. Acronyms: Angular gyrus (AG2), medial superior frontal gyrus (F1_2), inferior frontal sulcus (f2_2), pars opercularis (F3O1), pars triangularis (F3t), anterior insula (INSa2), anterior insula (INSa3), precentral sulcus (prec4), pre-superior motor areas (SMA2), pre-superior motor areas (SMA3), supramarginal gyrus (SMG7), anterior superior temporal sulcus (STS1; temporal pole), anterior superior temporal sulcus (STS2; anterior to Heschl’s gyrus), posterior superior temporal sulcus (STS3; posterior to Heschl’s gyrus), posterior superior temporal sulcus (STS4; posterior to Sylvian Fissure), superior temporal gyrus (T1_4), middle temporal gyrus (T2_3), posterior middle temporal gyrus (T2_4).

Figure 3 demonstrates that 0–cycles and 2–cycles, or rightward and leftward asymmetric structures, respectively, were identified in the language-related ROI. The density of asymmetries in each ROI depended on its size. For example, high correlations were observed between ROI volume and the mean density of 0–cycles and 2–cycles at the seventh and twelfth positions along the landscapes (0–cycle landscape position seven: r = 0.884, *p* = 1.165 × 10^−6^; 2–cycle landscape position 12: r = 0.839, *p* = 1.334 × 10^−5^). However, Figure 3 also shows that there was substantial variation across participants. That is, the size of the ROI determined the density of persistence pairs but not how much the density varied across participants within an ROI.

**Figure 4.**
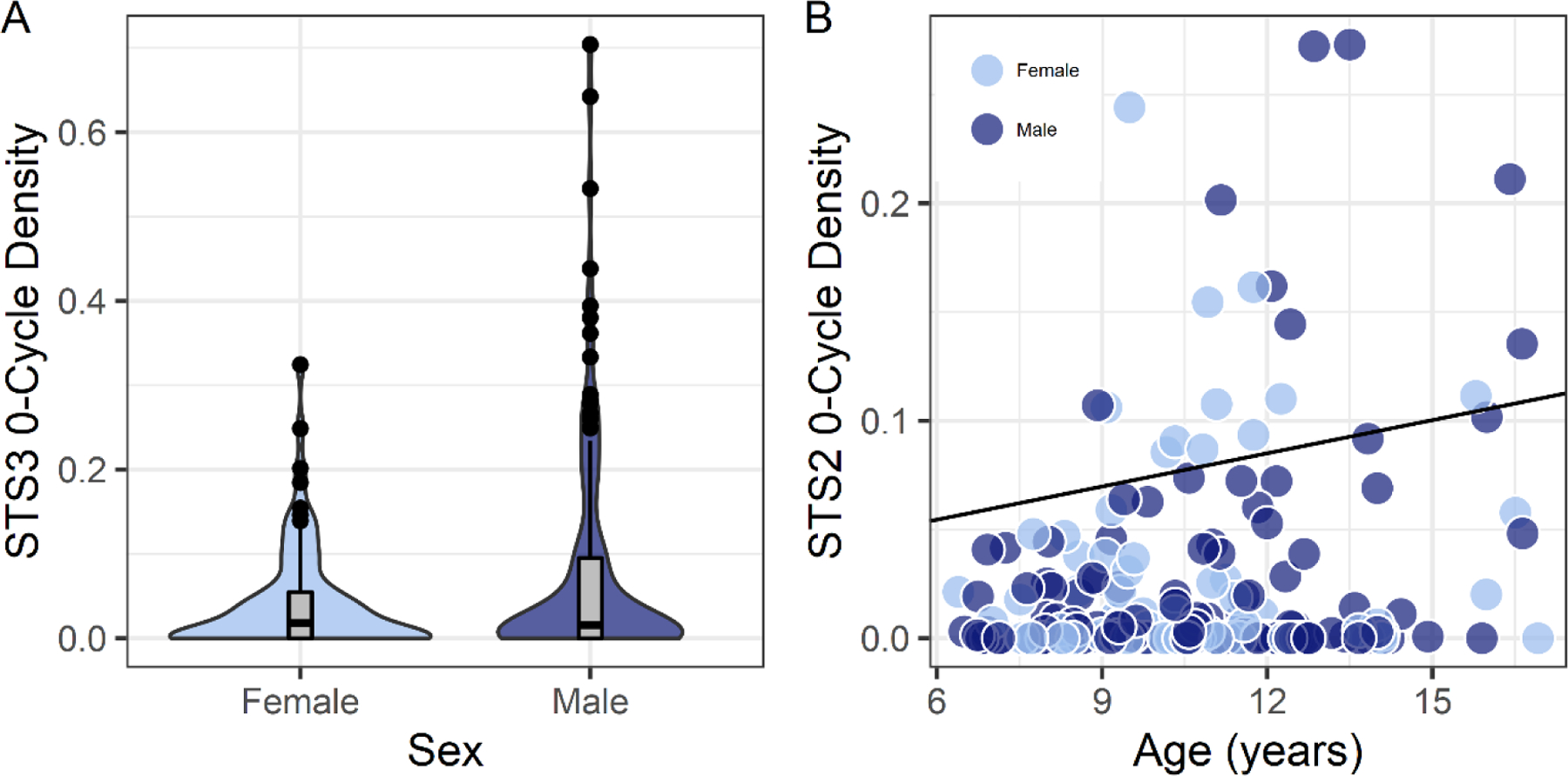
Rightward asymmetry structures (0–cycles) in the posterior and anterior superior temporal sulcus were more pronounced in males and with increased age, respectively. Permutation testing demonstrated that the sex effect in (**A**) and age effect in (**B**) were significant despite the appearance of a subset of cases contributing to the effects. y-axes: STS3 0–cycle Density landscape position 6; STS2 0–cycle density landscape position 5. Acronyms: Anterior superior temporal sulcus (STS2; anterior to Heschl’s gyrus), posterior superior temporal sulcus (STS3; posterior to Heschl’s gyrus).

The 0–cycle and 2–cycle density variances were explained in part by sex, age, and VIQ with small effect sizes. Results from the non-parametric linear regressions across language-related ROI are presented in Appendix A Supplementary Figure S2. This figure shows that some ROI exhibited consistently significant correlations with age and sex across landscape positions. Two of the largest and most spatially contiguous effects across a landscape are shown in Figure 4, where rightward asymmetries (0–cycles) in the posterior and anterior regions of the superior temporal sulcus were more pronounced in males and older children, respectively. These effects were not substantively affected when total brain volume was included in the regression models (e.g., sex and the posterior STS3 (landscape position 6): *t* = −3.071, *p* = 0.002, Cohen’s d = 0.44; total brain volume covaried: *t* = −2.719, *p* = 0.007, Cohen’s d = 0.39; e.g., age and the anterior STS2 (landscape position 5): *t* = 2.479, *p* = 0.014, Cohen’s d = 0.35; total brain volume covaried: *t* = 2.494, *p* = 0.013, Cohen’s d = 0.35)). VIQ associations with the asymmetry data included the posterior superior temporal sulcus where increased 0–cycle density was associated with higher VIQ scores (Supplementary Figure S2). However, VIQ associations across ROI were spatially sporadic and small in effect size.

The effect sizes for the landscape associations with sex, age, and VIQ were consistently larger than associations observed when using the mean asymmetry within the superior temporal sulcus ROI (sex and STS3: position 6, Cohen’s d = 0.44; ROI mean asymmetry: Cohen’s d = 0.34; age and STS2: position 5, Cohen’s d = 0.35; mean ROI asymmetry, Cohen’s d = 0.06). However, the differences in effect sizes between methods were not significantly different (sex: z = 0.485, *p* = 0.628; age: z = 1.444, *p* = 0.149).

**Figure 5.**
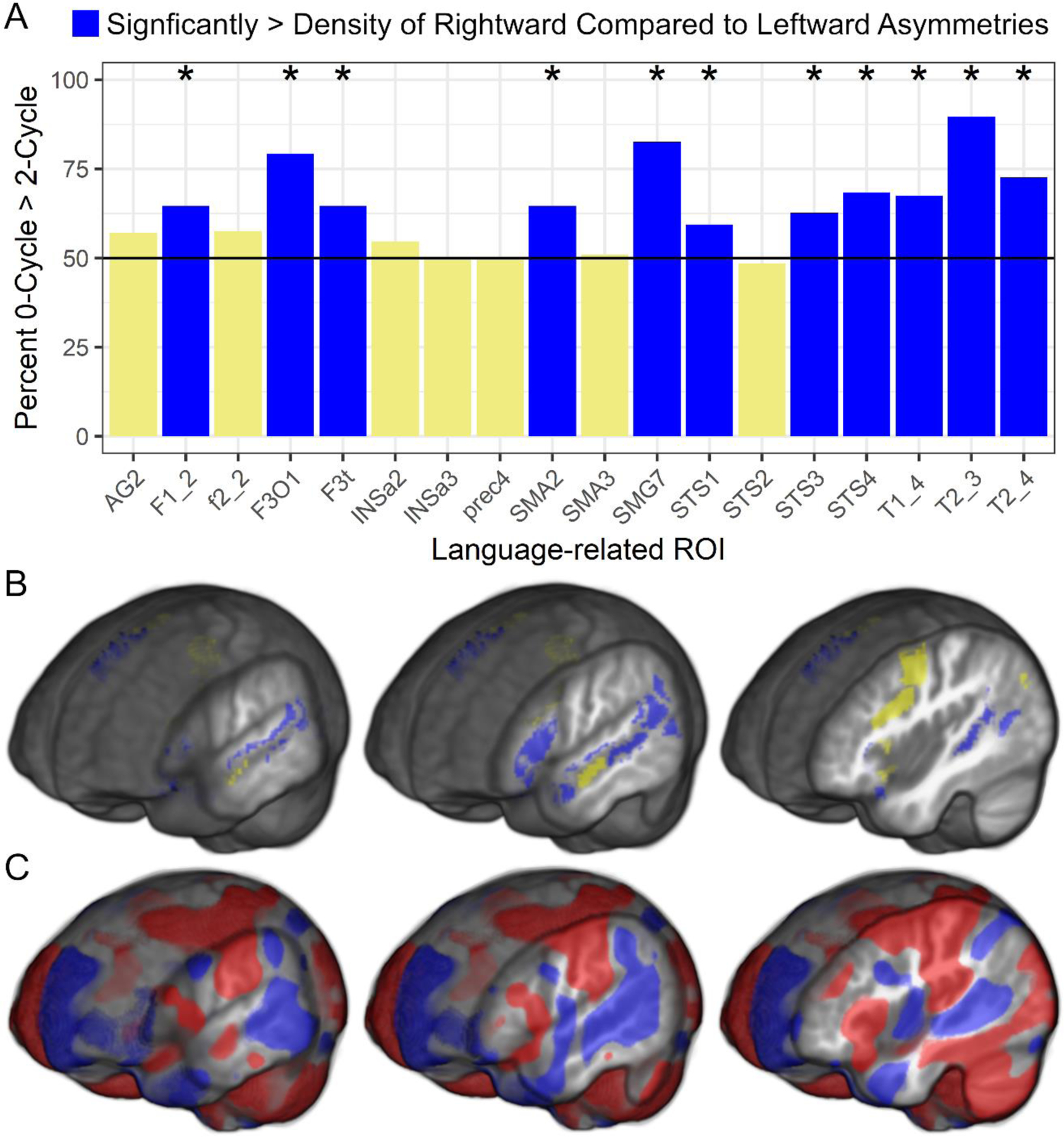
Topological and voxel-based asymmetries. (**A**) There was a consistently greater percentage of children with more rightward asymmetric structures (0–cycles) than leftward asymmetric structures (2–cycles) across language-related ROI. The y-axis is the percentage of children whose summed difference in 0–cycle and 2–cycle landscapes was greater than 0. * *p* = 0.05 to *p* < 0.000001. (**B**) This pattern of effects is presented for each of the ROI on the template image (blue: significantly rightward; yellow: non-significant). (**C**) The topological results largely overlap the whole brain voxel-based asymmetry results (TFCE FWE *p* < 0.05; blue: Rightward asymmetries; red: Leftward asymmetries). Acronyms: Angular gyrus (AG2), medial superior frontal gyrus (F1_2), inferior frontal sulcus (f2_2), pars opercularis (F3O1), pars triangularis (F3t), anterior insula (INSa2), anterior insula (INSa3), precentral sulcus (prec4), pre-superior motor areas (SMA2), pre-superior motor areas (SMA3), supramarginal gyrus (SMG7), anterior superior temporal sulcus (STS1; temporal pole), anterior superior temporal sulcus (STS2; anterior to Heschl’s gyrus), posterior superior temporal sulcus (STS3; posterior to Heschl’s gyrus), posterior superior temporal sulcus (STS4; posterior to Sylvian Fissure), superior temporal gyrus (T1_4), middle temporal gyrus (T2_3), posterior middle temporal gyrus (T2_4).

Finally, there were significant differences in the density of 0–cycle compared to 2–cycle asymmetries within each language-related ROI. Figure 5 shows that most participants had a significantly higher density of 0–cycles than 2–cycles across 11 of the 18 ROI and there were no ROI exhibiting a significantly higher density of 2–cycles than 0–cycles. Figure 5 also shows standard voxel-based asymmetry results to provide additional support for the rightward asymmetry topology findings. The 0–cycle > 2–cycle effects appeared to be consistent across age, sex, and VIQ as there were no significant relationships between these variables and the 0–cycle and 2–cycle difference metrics. That is, across children in this sample, there was a preponderance of rightward asymmetric structures within the SENSAAS defined cortical regions that exhibit leftward activity asymmetries during language tasks and this difference was not related to demographic or VIQ variables.

## 4. Discussion

The goal of this study was to characterize the three-dimensional topological asymmetries of language-related regions in a normative pediatric sample. Cortical regions that exhibit consistent patterns of leftward asymmetric activity in language studies exhibited both leftward and rightward structural asymmetries. These topological asymmetries were modestly influenced by demographic factors, particularly rightward superior temporal sulcus asymmetries that were more pronounced in males and older children, even after accounting for total brain volume. Across language-related ROI, there was a consistently higher density of rightward asymmetric structures than leftward asymmetric structures.

The current study differs from previous voxel-based asymmetry studies in an important way. Previous univariate asymmetry studies were designed to examine asymmetries within each brain voxel using spatially-dependent comparisons. The current study used a pure mathematics approach to characterize volumetric asymmetries that could span multiple voxels. Topological approaches are ideally suited for characterizing three-dimensional hyper-intensities and hypo-intensities. Here those structures represented hemispheric differences in the amount of volumetric displacement needed to warp the images to a symmetrical template. At the voxel level, these asymmetry data exhibit the same type of gray matter and white matter asymmetries that have been described previously [8] and appear to yield similar results to surface area asymmetries [12].

Figure 5 shows that the topological data exhibited a similar pattern of results to the voxel-based data, and the few spatially different results between approaches can be explained by the method of limiting the topological analyses to rightward asymmetries born within the ROI and leftward asymmetries that died within the ROI. That is, large deformation differences between hemispheres spanning multiple regions could have a death or peak asymmetry in a region outside of an ROI, which would not be included in the persistence diagram (e.g., large asymmetric deformations of primary somatosensory and motor cortex that extended into the language-related pre-central sulcus in Figure 5C). An advantage of this approach included a significant reduction in the number of variables compared to the voxel-based data. The topological approach also maintained the explicit relationship between the structural asymmetries and their range of voxel values in the persistence diagrams, which demonstrated considerable variation across participants and appeared to have greater sensitivity to individual differences than the mean asymmetry of voxels across an ROI.

### Demographic Influences on Structural Asymmetries.

The sex effects observed in this study are consistent with findings from voxel-based gray matter volume and surface area studies where males have exhibited more rightward asymmetry in the superior temporal sulcus than females [13,51]. The superior temporal sulcus typically exhibits a rightward asymmetry in voxel-based gray matter volume and deformation studies [8–12]. Here, the sex effect appeared to be driven by the greater prevalence of rightward asymmetries in males rather than more leftward asymmetries in females.

The age effects observed in this study also appear to be consistent with previous findings. For example, older age was associated with less leftward surface area asymmetry in the superior temporal sulcus [13]. In the current study, older children exhibited more rightward-asymmetric structures in the anterior superior temporal sulcus. This result in the current study was driven by a relatively small subset of participants (Figure 4). It seems likely that a large sample size and broad age range is necessary to observe this age effect. Together with the sex effects, demographic factors appeared to have a relatively limited association with topological asymmetries across the language-related regions.

### Verbal Comprehension.

VIQ associations with the topological asymmetries in language-related regions were relatively small and inconsistently observed across persistence landscapes (Supplementary Figure S2). These results are consistent with non-significant associations reported in studies of white matter diffusion and gray matter density asymmetries [25,52]. One explanation for these results is the limited range of VIQ in this normative sample compared to other studies where measures of VIQ were associated with structural asymmetries (e.g., [9]).

### Limitations.

Functional asymmetry data were not available for this study. Thus, it was not possible to determine the extent to which the sample exhibited rightward asymmetries in language function that might map to their rightward structural asymmetries. However, given the relatively large sample size of children with relatively average to above average VIQ, it seems likely that the sample is representative of the normal population exhibiting leftward language laterality in brain activity. A related limitation is the absence of a quantitative handedness measure in this study. Variation in hand dexterity and preference may have contributed to variation in the topological asymmetry measures, and there is some evidence from a meta-analysis for a handedness effect on structural asymmetries [13]. However, that same study also demonstrated no effect of handedness on cortical thickness asymmetries in a very large sample size. Finally, we also were unable to determine the extent to which variation in topological asymmetries was influenced by multi-lingual children [53], which, along with other skills such as musicianship, seem important areas of future topological study.

## 5. Conclusions

Cerebral asymmetries are multi-dimensional and exhibit significant variability across humans [54], including increased variation within local specific regions that can be uncoupled to variation in other regions in comparison to non-human apes [55]. Here we demonstrated rightward and leftward asymmetric structures within locally specific cortical regions that typically exhibit leftward asymmetric activity during language tasks. These structural asymmetries exhibited patterns of variation with sex and age that were largely consistent with previous voxel-based studies, particularly studies where modulated gray matter (i.e., gray matter volume) and surface area asymmetries were examined. The most unexpected result from this study was the greater density of rightward compared to leftward asymmetric structures in language-related regions. In addition to the anterior insula that exhibits leftward asymmetry across structural studies and appears to explain some of the variance in lateralized language expression [14–16], perhaps it is the combination of structural asymmetries that underlies asymmetrical patterns of activity for language expression and reception.

## Figures and Tables

**Table 1.** T1-weighted image parameters from the 10 study sites.

Site	Manufacturer	Field Strength (T)	Image Dimension (mm)	Slice Thickness (mm)	TR (msec)	TE (msec)	Flip Angle (deg)
1	Siemens	1.5	256 × 256 × 160	1.60	25.00	4.60	30
2	Siemens	3.0	176 × 240 × 256	0.90	2250.00	3.96	9
3	Siemens	3.0	128 × 256 × 256	1.33	6.60	2.90	8
4a	GE	1.5	124 × 256 × 256	1.20	11.10	2.20	25
4b	GE	1.5	124 × 256 × 256	1.40	11.10	2.20	25
5	Siemens	3.0	160 × 256 × 256	1.00	1600.00	3.37	15
6	Philips	1.5	170 × 256 × 256	1.00	8.02	3.69	7
7	GE	1.5	181 × 217 × 181	1.00	6.00	63.00	–
8	Siemens	1.5	160 × 256 × 256	1.00	2000	3.65	8
9	GE	3.0	256 × 256 × 124	1.20	9.00	2.00	15
10	Philips	3.0	256 × 256 × 120	1.10	10.00	6.00	8

**Figure A1.**
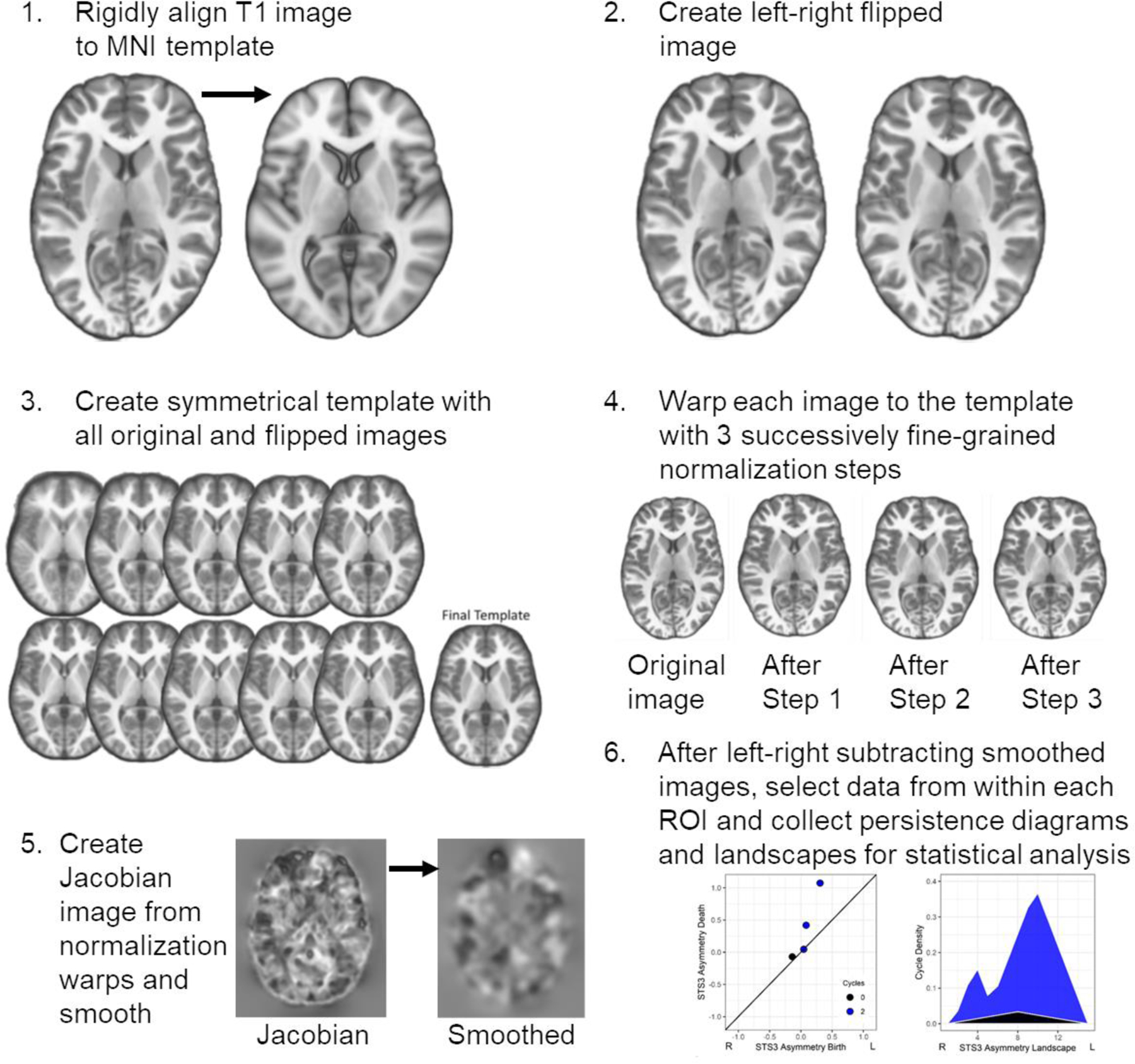
Overview of the image processing steps used to generate structural asymmetry images based on voxel-level volumetric displacement data from warping each T1-weighted image to a study-specific template.

**Figure A2.**
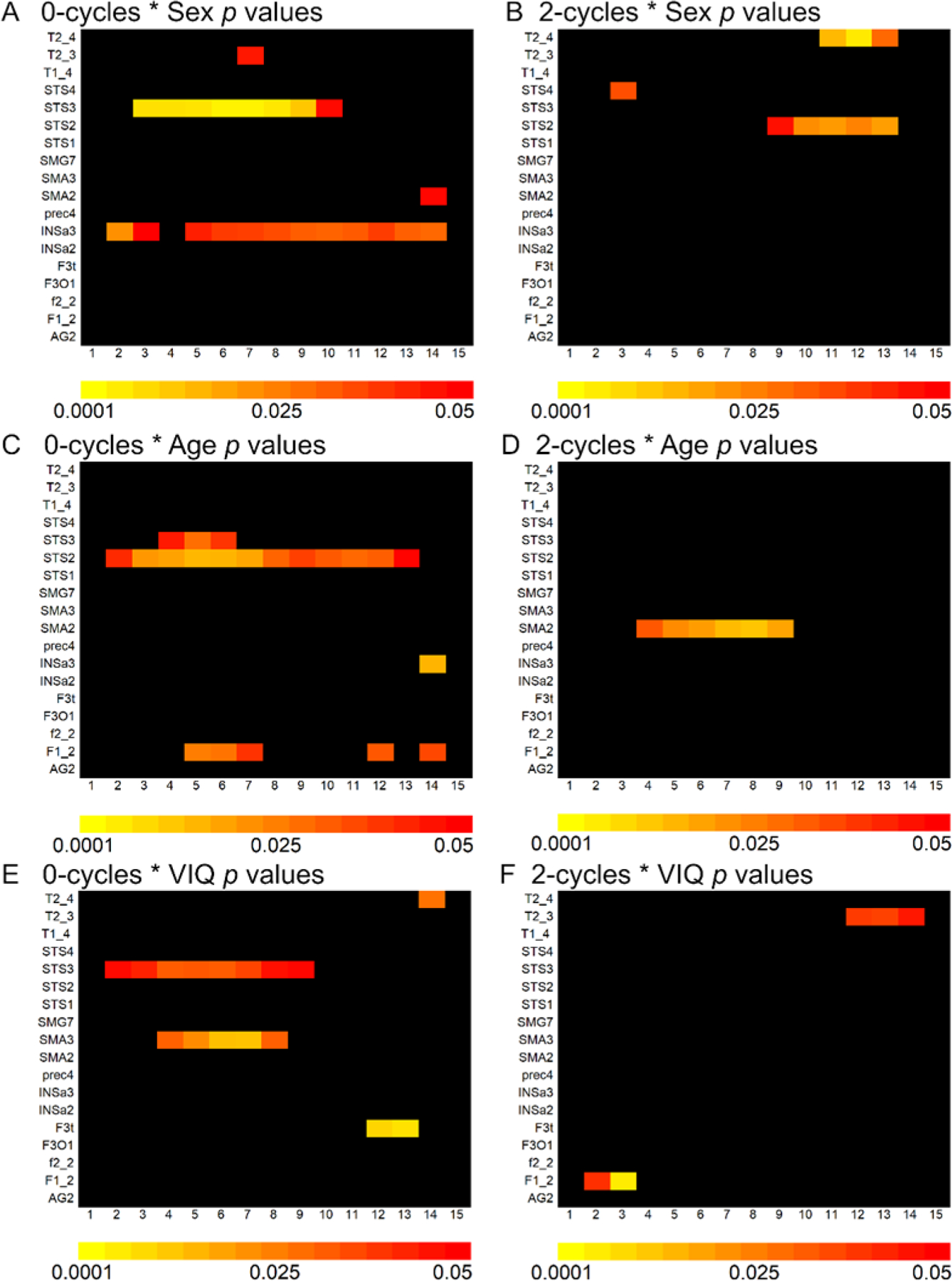
The spatial distribution of landscape associations with sex, age, and VIQ shown in for 0–cycles (**A**,**C**,**E**) and 2–cycles (**B**,**D**,**F**), respectively. Most notable was the greater density of rightward asymmetries in the posterior superior temporal sulcus (STS3) in males compared to females as shown in (**A**) as well as the greater density of rightward asymmetries in the anterior superior temporal sulcus older children (STS2) as shown in (**C**). These non-parametric linear regression results, which included sex, age, VIQ, and research site in the analyses, are color coded for associations exhibiting *p* < 0.05 associations. Acronyms: Angular gyrus (AG2), medial superior frontal gyrus (F1_2), inferior frontal sulcus (f2_2), pars opercularis (F3O1), pars triangularis (F3t), anterior insula (INSa2), anterior insula (INSa3), precentral sulcus (prec4), pre-superior motor areas (SMA2), pre-superior motor areas (SMA3), supramarginal gyrus (SMG7), anterior superior temporal sulcus (STS1; temporal pole), anterior superior temporal sulcus (STS2; anterior to Heschl’s gyrus), posterior superior temporal sulcus (STS3; posterior to Heschl’s gyrus), posterior superior temporal sulcus (STS4; posterior to Sylvian Fissure), superior temporal gyrus (T1_4), middle temporal gyrus (T2_3), posterior middle temporal gyrus (T2_4).
